# Vaginal and vestibular electrical resistance as an alternative marker for optimum timing of artificial insemination with liquid-stored and frozen-thawed spermatozoa in sows

**DOI:** 10.1038/s41598-023-38803-5

**Published:** 2023-07-26

**Authors:** Dannielle Glencorse, Christopher G. Grupen, Roslyn Bathgate

**Affiliations:** Sydney School of Veterinary Science, Faculty of Science, The University of Sydney, Sydney, Australia

**Keywords:** Biological techniques, Physiology

## Abstract

Development of a pen-side test to objectively determine the ideal time for artificial insemination (AI) in the sow would save producers time and money. Current processes rely on identification of oestrus via subjective behavioural and physiological markers that are indicative of high blood oestrogen concentrations. This study attempted to use measurements of electrical resistance (ER) in the cervical mucus to pinpoint timing of AI accurately enough to lead to equivalent pregnancy rates as a natural mating. Thirty-six sows were divided into 3 groups and observed for signs of oestrus. Seven sows did not show any oestrus behaviour and were excluded from the study. The remaining 29 sows were inseminated via natural mating and conventional oestrus detection (n = 14), or inseminated artificially with either liquid-stored semen (n = 8) or frozen-thawed semen (n = 7) according to timing indicated from electrical resistance measurements in the vagina and vestibule. Sows that were artificially inseminated on the basis of the electrical resistance readings had a lower pregnancy rate (P = 0.034) and less piglets born alive per litter (P < 0.05) than those that were naturally mated according to a conventional oestrus detection regime. However, the pregnancy rate and total piglets born alive did not differ between the two groups that underwent artificial insemination. Change in electrical resistance in the vagina has the potential to accurately predict ovulation timing, but more work is required to refine the timing of AI in relation to the readings before the technique can be adopted by industry.

## Introduction

Artificial insemination (AI) in sows is a process that requires multiple doses of semen, delivered daily due to the uncertainty in accurately predicting ovulation^1,2^. Conventional AI in sows involves a labour-intensive monitoring program for detecting oestrus by visual observation of behavioural indicators such as flank nosing, snout contact, urogenital sniffing, mounting of other sows and the presence of standing heat in response to back pressure^3^. Physical changes such as vulval redness and swelling or discharge from the vulva are also used in oestrus detection programs^4^. These observations are time-consuming, often variable between animals and require substantial training due to their subjective nature, leading to difficulty in identifying these changes^5^. More accurate methods focused on detecting ovulation instead of the general oestrus period would allow stock-people to achieve more consistent insemination outcomes^6^. Additionally, an objective tool could provide the additional benefit of reducing the number of semen doses if the exact moment of ovulation can be predicted^7,8^.

Inseminations with frozen-thawed spermatozoa require more precise detection of the specific timing of ovulation rather than just identifying the oestrus period^9,10^. Frozen-thawed spermatozoa has a post-thaw lifespan of approximately 16–20 h less than liquid-stored spermatozoa^4^. Liquid-stored spermatozoa can survive in the female reproductive tract for up to 24 h while frozen-thawed spermatozoa have a limited lifespan of approximately 4–8 h^8,9,11^. The reduced lifespan of frozen-thawed spermatozoa causes a reduction in the motility and affects DNA integrity leading to substantial effects on farrowing rate and litter size^12,13^. These issues lead to a shortened window of opportunity for semen deposition and therefore an increased requirement for oestrus detection labour when implementing frozen-thawed semen artificial insemination (FAI) programs^14,15^. One potential method for overcoming these issues is through monitoring of electrical resistance levels in the vagina and vestibule^16–18^.

Electrical resistance (ER) is a value that quantifies the opposing force that impedes the flow of electrical current through an object^19^. The resistance of an object is determined by the size and material each object is made from^20^. A positive or negative charge is produced by each object and this can be measured by introducing an electrical current to the surface of the object^21^. This concept can be applied to the measurement of mucosa epithelium conductivity^22,23^. Electrodes implanted in the reproductive tract of cows determined a reduction in the electrical resistance during the oestrus period^21^. This is because an increase in the vascularity of tissues in the reproductive tract leads to fluctuations in the electrical potential of the surface^24^. Vaginal ER has been monitored throughout oestrus and has been shown to be correlated with increasing concentrations of oestrogen in the Okinawan native Agu pig^17^. A profile of vaginal ER in sows identified a decrease to the minimum level during the early stages of the follicular phase, followed by an increase to peak levels in the early luteal phase^17,22^. These vaginal ER changes are well correlated with fluctuating oestrogen and LH concentrations^25^. Comparable conception rates of 85% have been obtained using conventional oestrus detection and vaginal ER-predicted oestrus detection, when double inseminations of liquid-stored spermatozoa were performed^26^.

Greater FAI fertility may be possible by identifying biological markers associated with ovulation instead of using conventional behavioural observation to predict insemination timing^10,15^. Farrowing rates of 80% and litter sizes of 10 can be obtained when frozen-thawed spermatozoa are inseminated within 4–8 h of ovulation by experienced technicians^27,28^. However, these fertility results are only achievable when observations occur at 4-hourly intervals^29^ Frozen-thawed semen AI is not commonly used in the pig industry due to the requirement for frequent oestrus observation and the relatively low fertility obtainable compared with liquid-stored semen AI^5^.

This study aims to determine if the electrical resistance readings in the vaginal and vestibular regions are capable of detecting the correct timing for artificial insemination. This will be done by comparing fertility outcomes from sows bred naturally using conventional oestrus detection with sows artificially inseminated using ER to determine timing of insemination.

## Materials and methods

Chemicals were sourced from Sigma-Aldrich (St Louis, MO, USA) unless otherwise stated. All work was carried out in accordance with the University of Sydney’s Research Code of Conduct and the Australian Code for the Responsible Conduct of Research^30^. The methods are reported in accordance with ARRIVE guidelines^31^.

### Animal management

The methods involved in this study were approved by the Animal Ethics Committee at The University of Sydney (2013/5942), under the guidance of the Australian Code for the care and use of animals for scientific purposes^32^. Data were collected from Large White, Landrace and Duroc crossed sows (n = 36) at The University of Sydney piggery. Boars were housed in single pens, allocated ad libitum water and a low energy ration. Sows were batch housed in large deep litter pens in groups prior to parturition in farrowing crates with ad libitum water and a high energy lactation ration. Body condition score was recorded upon entry to the farrowing house. They were moved from farrowing crates to batch housing in groups of 4–6 sows based on a pen size of approximately 14 m^2^.

### Experimental design

Sows were randomly split into three treatment groups based on different proposed oestrus detection and insemination protocols; control/natural boar mating (n = 14), liquid-stored AI (n = 8) and frozen-thawed AI (n = 7). The natural mating group underwent traditional behaviour-based detection of oestrus followed by joining with a breeding boar for a double insemination protocol at 0 and 24 h after the first recorded instance of behavioural oestrus. The liquid-stored and frozen-thawed groups had behavioural oestrus recorded but were inseminated according to vaginal ER-detected oestrus protocols as demonstrated in Yamauchi et al.^17^. A double dose protocol was used with inseminations conducted 0 and 24 h and 12 and 24 h after a detected increase in vaginal ER for liquid-stored and frozen-thawed treatments, respectively.

**Figure 1:**
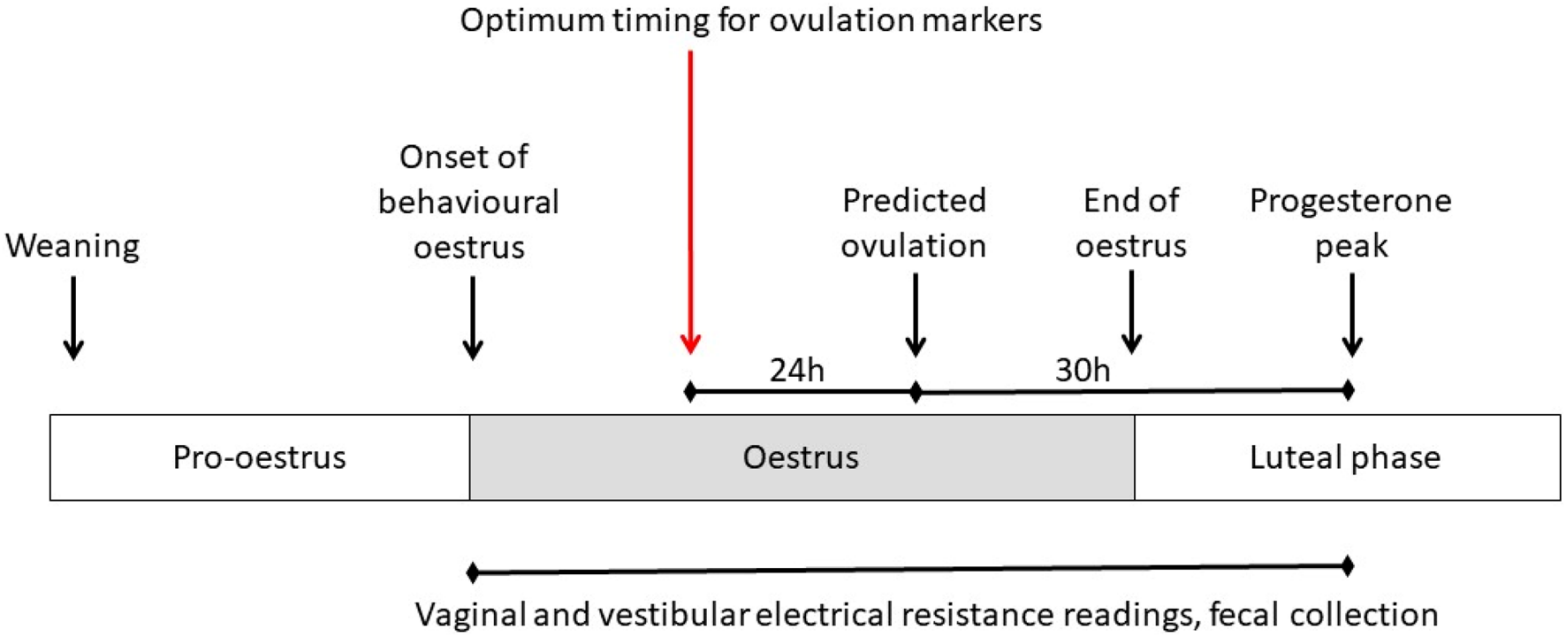
Schematic of the timings of examinations conducted during oestrus in sows to identify if electrical resistance is an effective physiological marker of optimum insemination timing. Day 0 is the first time-point in the oestrous cycle and is defined as the first instance of observed behavioural oestrus. The horizontal arrows lead to time-points that exist at an appropriate time relative to oestrus events and hence will enable identification of the physiological markers that could predict ovulation accurately.

The four time points examined were the onset of behavioural oestrus, 24 h after the onset of behavioural oestrus, the estimated instance of ovulation as predicted by faecal hormone concentrations, and 24 h prior to predicted ovulation (Fig. 1). In all treatment groups, an individual sow was deemed to be in oestrus if the standing heat response was observed following application of back pressure in the presence of a mature boar. The first instance of behavioural oestrus or the onset of oestrus was defined as the first observation of standing heat in response to back pressure from the stockperson. The instance of predicted ovulation was determined by identifying the concentration of progesterone obtained in faecal collections. This time-point was defined as 30 h prior to the peak concentration of progesterone, which accounted for dispersal of hormones in the faeces and faecal transit through the digestive tract as demonstrated by Snoj et al.^33^.

### Electrical resistance

Electrical resistance of the reproductive tract was monitored twice each day (07:00/15:00) using a Draminski resistance probe (Draminski-Electronics, Olsztyn, Poland). The probe was disinfected in an iodine solution and dried before and after use in each sow. The vulva was cleaned, and the probe inserted into the vagina on an upward angle of 20–35° until resistance from the cervix was encountered. The probe was operated as per manufacturer instructions^34^. The electrical resistance values for the epithelium of the vagina and the posterior epithelium of the vestibule were recorded.

### Oestrus detection and insemination protocols

Oestrus detection was recorded from 3 days after weaning until 2 days after the last instance of behavioural oestrus. Sows were observed for 30 mins twice a day (07:00/15:00) to identify oestrus behaviours including standing heat, flank nosing, snout contact, sow-to-sow mounting and urogenital sniffing. Individual sows were deemed to be in oestrus if the standing heat response was observed following application of back-pressure in the presence of a mature boar. The liquid-stored and frozen-thawed treatment groups underwent vaginal ER-detected oestrus. This procedure involved daily recording and monitoring of ER from the vagina and vestibule, with oestrus defined as the first increase in ER following basal level readings. The basal level was defined as the lowest readings taken in the days prior to onset of behavioural oestrus.

Control sows were joined with a sexually mature boar in a designated mating area at 0 and 24 h after standing heat was first detected, with assistance provided to boars during copulation. Both liquid-stored and frozen-thawed treatment sows were moved to a stall alongside a mature boar and inseminated according to the treatment using a Melrose style catheter to deposit semen into the cervix (Minitube, Tiefebach, Germany).

### Liquid-stored spermatozoa

Semen was collected from the boars using the gloved hand method into a filtered collection bag held in a pre-warmed 37 °C thermos flask. Only ejaculates displaying total motility greater than or equal to 80% were used. Semen was extended with Androstar Plus (Minitube, Germany) to make AI doses of 3.5 × 10^9^ total sperm in 80 mL and cooled to 15 °C over 2 h. The spermatozoa were maintained at this temperature for 3 and 4 days until use.

### Frozen-thawed spermatozoa

The sperm-rich fraction of semen was collected from a boar using the gloved hand method into a filtered collection bag held in a pre-warmed 37 °C thermos flask^35^. Only ejaculates with motility of 80% or greater were used. The semen was extended (1:3 semen:Androstar Plus) and cooled to 15 °C over 2 h. The extended semen was centrifuged at 800 × *g* for 20 min at 15 °C and the resultant pellet was resuspended in cooling extender (80% v/v of 11% w/v lactose solution with 20% egg yolk) to give a concentration of 1.5 × 10^9^ spermatozoa/mL. The suspension was cooled to 5 °C over 1.5 h, when a freezing extender (89.5% cooling extender, 1.5% (v/v) Equex STM (IMV, L’Aigle, France) and 9% glycerol) was added to give a final concentration of 1 × 10^9^ spermatozoa/mL. The spermatozoa were frozen in 0.5mL straws (IMV, L’Aigle, France) using a programmable freezer with the following settings: 5 °C to – 6 °C at – 3 °C per min held at – 6 °C for 1 min and cooled from – 6 °C to – 140 °C at – 50 °C per min. Samples were immediately plunged into liquid nitrogen (LN_2_) for storage until use. The straws were thawed by agitating in a 37 °C water bath for 40–60 s until liquefied and warm to touch. The 15–18 straws that were thawed yielded a single dose of 3.5 × 10^9^ motile spermatozoa for insemination and AndrostarPlus was slowly added to make a final volume per dose of 80 mL. Frozen-thawed samples were only used if progressive motility was greater than or equal to 40%.

### Faecal progesterone assay

Faecal samples were collected twice daily from each sow and stored at – 80 °C until further processing. Each sample was dried at 65 °C overnight or until dried thoroughly. The samples were crushed into a fine dust and large contaminants such as straw were removed. Samples were extracted using ethanol or ethyl acetate at a rate of 1 mL/0.1 gram (Sigma-Aldrich 24102-1L-R, > 99.8%). All samples were mixed overnight and centrifuged at 4200 × *g* for 15 mins. A 500 µL volume of supernatant was transferred to a clean tube for evaporation in a SpeedVac vacuum oven. The dried extracted samples were frozen and stored at – 20 °C in a desiccator prior to use in a DetectX^®^ Progesterone ELISA Kit (Arbor assays, Michigan, USA).

Extracted samples were mixed with 100 µL of ethanol followed by 400 µL of assay buffer (1:5 dilute AB concentrate: deionised water). The samples were vortexed three times and allowed to sit at room temperature for 5 mins and diluted with 1–5 mL of ethanol and assay buffer. The reconstituted diluted samples were run in duplicate in a 96-well assay plate. A 25 µL volume of progesterone conjugate (DetectX^®^ progesterone-peroxidase conjugate in stabilising solution, Arbor Assays) and 25 µL volume of progesterone antibody (DetectX^®^ mouse progesterone specific monoclonal antibody, Arbor Assays) were added to each well and mixed thoroughly. A plate shaker was used to mix the contents of the wells for 2 h. The plate was aspirated five times with wash buffer (1:20 dilution of wash buffer concentrate to deionised water) before adding 100 µL of TMB substrate (3,3’,5,5’—tetramethylbenzidine, Arbor Assays) to each well. The samples were incubated for a further 30 mins before adding 50 µL of stop solution. The optical density of each sample was recorded using a plate reader (4PLC software) at a wavelength of 450 nm. The progesterone concentration was calculated based on a standard curve using 4PLC software.

### Fertility assessment

Sows were assessed for return to oestrus 21–25 days post-insemination. Transcutaneous ultrasound (SonoSite Vet 180 Plus) was performed 35 days after insemination and conception rates were recorded. The farrowing rate, litter size, piglets born alive and stillborn piglets were recorded post-parturition.

### Statistical analysis

Statistical analyses of the data were completed using the R statistical software package^36^. The non-return rates and pregnancy rates were assessed using a Generalised Linear Mixed Model. This model indicates the probability of return to oestrus based on the effects of insemination type (natural mating, liquid-stored or frozen-thawed). A REML Linear Mixed Model was used to analyse the correlation between vaginal ER and timing within the oestrous cycle. Effect of probe location was assessed using correlation analysis in the form of Pearson’s tests. A level of P < 0.05 indicated a statistically significant result for all tests.

### Ethics approval and consent to participate

Ethics approval was granted for use of animals by the University of Sydney Animal Ethics Committee.

## Results

Of the 36 sows included in this study, 29 demonstrated behavioural oestrus and were inseminated according to the treatment allocation. Reproductive data of the cycling sows used in the study are shown in Table 1. The sows that did not undergo an oestrus event and therefore did not experience an insemination were removed from the experiment.

**Table 1:** Reproductive data of the cycling sows included in the study.

Sample sow data	Mean ± S.E.M	Range
Sample size	29	
Parity	2.41 ± 0.27	1–7
Body condition score	3.12 ± 0.07	2.5–4
Lactation length		
Wean-oestrus interval (days)	4.6 ± 0.42	2–7
Length of behavioural oestrus (h)	58.3 ± 0.48	49–68
Estimated ovulation based on behavioural oestrus (h)	37.0 ± 0.51	32–42

**Figure 2:**
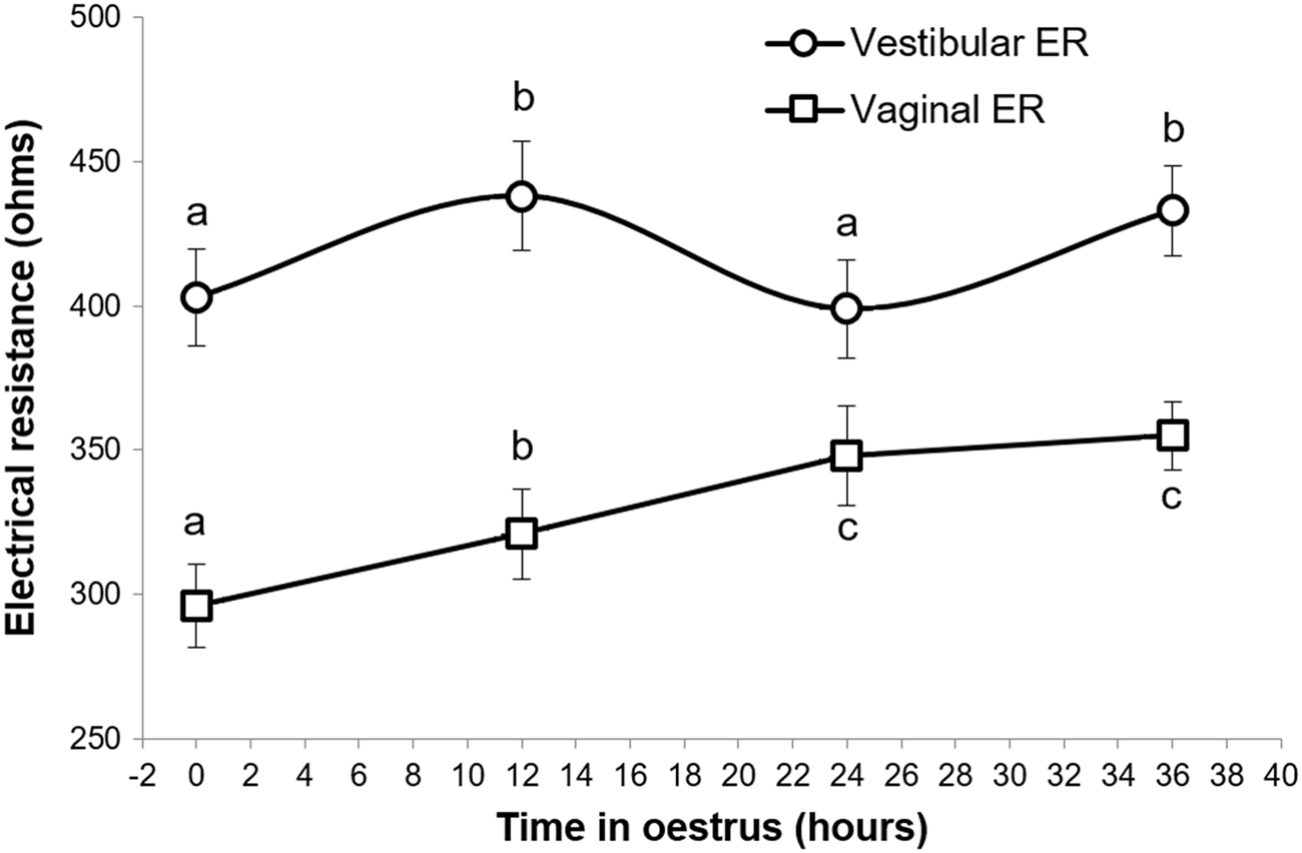
Mean (± S.E.M) electrical resistance recorded at two locations within the reproductive tract (vestibule and vagina) during the oestrus cycle. Time points are relative to the onset of behavioural oestrus detected by the presence of standing heat in the presence of a boar. The last data point coincides with predicted ovulation, which was defined as 30 h prior to the highest detected faecal progesterone concentration. Superscripts indicate statistical differences between time-points within one location (P < 0.05).

Electrical resistance changed throughout the oestrous cycle (Fig. 2). A cyclic change was observed in both vaginal and vestibular electrical resistance with levels increasing from the time of observed behavioural oestrus. This increase continued in the vaginal readings, but the vestibular readings declined 24 h after oestrus onset, then rose again (P = 0.023). Both resistance measurements gradually decreased from the point of predicted ovulation until plateauing in the early luteal phase (Fig. 2). Vaginal electrical resistance was positively correlated with vestibular electrical resistance (P < 0.001; r = 0.655). However, the variation in the vestibular measurements was greater than that of the vaginal measurements.

### Fertility

The length of oestrus was consistent amongst the three treatment groups with no significant differences identified. All sows that were deemed to have conceived resulted in a successful pregnancy (Table 2). The conception rates and farrowing rates for the liquid-stored and frozen-thawed inseminations (both performed at timings determined by vaginal ER) were significantly lower than those for the control sows (inseminated according to commercial oestrus detection protocols). The ER predicted inseminations resulted in farrowing rates of 72.73% which was significantly lower than the control sows (P = 0.034). Also, the number of piglets born alive per litter was less in the liquid-stored and frozen-thawed groups compared with the control group (P < 0.05). Similar conception rates, farrowing rates and numbers of piglets born alive per litter were obtained for the liquid-stored and frozen-thawed inseminations (P > 0.05). The number of stillborn piglets per litter did not differ among the groups (P > 0.05).

**Table 2:** Oestrus, conception and farrowing rates obtained from control sows inseminated naturally using conventional oestrus detection, and sows inseminated with liquid-stored and frozen-thawed semen using ER to determine the timing of insemination.

Treatment group (n)	Oestrus length (h)	Conception rate (%)	Farrowing rate (%)	Born alive	Stillborn
Conventional oestrus detection and natural mating (14)	52.5 ± 2.2	90.9%^a^	90.9%^a^	12.1±1.6^a^	0.30
AI of chilled sperm after oestrus detection with ER (8)	54.5 ± 2.1	72.7%^b^	72.7%^b^	9.5±1.6^b^	0.13
AI of frozen-thawed sperm after oestrus detection with ER (7)	57.5 ± 3.6	66.7%^b^	66.7%^b^	7.7±1.1^b^	0.17

The superscripts indicate a statistical difference of P < 0.05 between insemination types within each column.

## Discussion

This study found correlations between vaginal and vestibular electrical resistance readings and the timing of ovulation, as predicted by faecal hormone analysis, suggesting that ER-detected oestrus is a promising ovulation detection tool for predicting the optimum AI timing. This is possible as elevated secretions of cervical mucus are associated with increased oestrogen production in the reproductive tract prior to ovulation which contributes to the elevated conductance of mucosal tissues^37^. These results support previous studies which indicated that changes in the readings from ER probes could be used to predict optimum insemination time and confirm that AI should be conducted at the first instance of vestibular ER increasing relative to the basal level, followed by a second insemination 24 h later^16,17,25,34^.

The correlation between vaginal and vestibular ER and the peri-ovulatory period was found despite one of the study limitations being that ovulation time was gauged by fecal progesterone assay. This was used rather than other options that may have given greater precision in the timing of ovulation, such as ultrasonography or detection of the luteinising hormone surge as a less invasive method that has been previously shown to accurately estimate ovulation timing^38^.

The lowest vaginal ER was identified at the onset of behavioural oestrus while the lowest vestibular ER was recorded 24 h after the onset of oestrus, estimated to be 24–30 h prior to ovulation and so ideal for calculating insemination timing. This is supported in part by previous studies^18,39^ where the ER was seen to decrease prior to ovulation. However, these other studies did not observe a difference in ER pattern between vaginal and vestibular readings^39,40^. This may be due to the fact that the previous study inserted the probe only 2–3 cm past the vulva for the vaginal readings compared with the approximately 10 cm insertion depth used here. Other authors have found that ER readings taken in the vagina are variable and less reliable than those taken in the vestibule^41^, which may also explain the disparate findings between studies. Variations between the measurements taken in the vagina and vestibule may be due to the composition or volume of the cervical mucus present within these locations^40^, which vary in relation to oestrogen concentration^16,25^. Parity and sow weight introduce some variation in the size of the vestibule^42^. The process of parturition can cause damage to the reproductive tract, particularly in multiparous sows, which may lead to inconsistent surface-probe contact and increased ER variation.

Typical commercial insemination protocols using behaviour-based oestrus detection to determine the optimum timing for insemination result in conception rates of 85–100% and 40–90% for liquid-stored and frozen-thawed AI respectively^8,10,14,43^. Any replacement oestrus detection method should be capable of obtaining comparable or improved results in order to warrant adjustments to existing management protocols. The current study observed a reduction in conception rates for liquid-stored AI but maintained acceptable rates for the frozen-thawed treatment group when compared to conventional AI outcomes reported previously^44,45^. Using ER to predict when AI should be conducted caused the timing of AI to slightly differ compared with other studies, which may have led to the lower fertility rates rates for the liquid-stored AI group. Reduced oestrus length is associated with earlier ovulation^46^ and this variation in the point of ovulation within the oestrus period may have contributed to the lower than average conception rates in the liquid-stored group. Using ER to identify the optimum AI timing may be difficult in these sows as a reduced farrowing length would impact on the timing of ER changes, causing fluctuations to occur faster and making detection difficult. When focusing on the frozen-thawed group, conception rates were comparable with those obtained commercially. However, as the expected lifespan of frozen-thawed semen is 4–8 h post-thaw, an ER oestrus detection protocol could lead to an absence of viable spermatozoa within the reproductive tract at the time of ovulation^28^. The current study performed frozen-thawed AI at 12 and 24 h intervals after a detected increase in vaginal ER (equating to 24 and 36h after the start of oestrus), leading to a conception rate of 67%. Previously, Yamauchi et al.^17^ reported a conception rate of 41% when fresh semen was inseminated at 24 and 34 h after an increase in vaginal ER in the Okinawan native Agu pig. Differences in the reproductive physiology of the Agu and commercial domestic pig breeds likely explain the disparate findings. Given the short fertilizing lifespan of frozen-thawed semen and that ovulation occurred about 37 h after the onset of behavioural oestrus, our findings indicate that the insemination at 24 h after the first detected ER increase (or about 36 h after the onset of oestrus) provided the fertile sperm required for conception. Therefore, using this insemination timing, the described procedure could be implemented commercially within the typical workday of 8 h to enable the benefits of a single frozen-thawed AI to be achieved. Studies refining the timing of AI with frozen-thawed semen are needed to realise the potential of this technology.

These results show that a minimum of two resistance measurements each day are required to accurately detect a change in conductance. Collection of this objective information is a short process with a labour requirement of 10–20 s per sow^34^, less than the 10–15 min per animal for conventional detection methods^46,47^. Additionally, this technique can be conducted without moving the sow to the presence of a boar, as the measurement encapsulates the physiological changes that are driven by hormonal changes throughout the oestrous cycle instead of momentary behavioural fluctuations that are stimulated in the sow in anticipation of copulation^48,49^. While these factors highlight the potential effectiveness of ER-detected oestrus for precision oestrus detection, the technology requires further refinement to become a commercial reality. For example, consideration of biosecurity when sharing equipment between animals and identifying the correct timing of insemination in relation to observed ER changes.

## Conclusions

In conclusion, insemination at the first detected increase in vaginal ER, relative to basal levels, resulted in conception and farrowing rates that were similar for liquid-stored and frozen-thawed spermatozoa. However, these rates were significantly lower than those obtained using conventional oestrus detection and natural mating. This suggests that the technology has commercial potential, but timing of insemination relative to basal ER readings should be confirmed under differing circumstances such as variable lactation length, weaning to oestrus intervals and sow parity.

## Data Availability

The datasets used and/or analysed during the current study are available from the corresponding author on reasonable request.

## Abbreviations

AI: Artificial insemination
ER: Electrical resistance
FAI: Frozen-thawed semen artificial insemination
